# Factors facilitating and hindering counselling about generic substitution and a reference price system in community pharmacies - a survey among Finnish dispensers

**DOI:** 10.1186/s12913-022-08477-2

**Published:** 2022-09-07

**Authors:** Riikka Rainio, Riitta Ahonen, Elina Lämsä, Johanna Timonen

**Affiliations:** School of Pharmacy, Social Pharmacy, Faculty of Health Sciences, Kuopio Campus, University of Eastern Finland, PO Box 1627, FI-70211 Kuopio, Finland

**Keywords:** Generic substitution, Interchangeable medicines, Reference price system, Patient counselling, Community pharmacy, Benefits, Problems

## Abstract

**Background:**

Pharmacies play the key role in implementing generic substitution (GS) and counselling customers about it. This study aimed to explore dispensers’ perceptions of the factors that facilitate or hinder counselling customers on GS and the reference price system (RPS) in community pharmacies. It also studied dispensers’ opinions about the benefits and problems of these systems and discusses them from the counselling point of view.

**Methods:**

A postal survey was conducted among Finnish community pharmacy dispensers in spring 2018. The research questions were studied through open-ended questions and analyzed both qualitatively and quantitatively. The questions were analyzed first with inductive content analysis by two researchers independently. The responses were encoded and categorized according to the analytical framework, which was inductively developed alongside the analysis. The categorized responses were further analyzed using frequencies and percentages.

**Results:**

The response rate was 50.8% (*n* = 498). Of the respondents, 75.9% reported factors that facilitated counselling about GS and RPS. The most commonly mentioned factors included customers’ characteristics (36.5%), the information systems used in the pharmacy (28.3%), and the features of interchangeable medicines (21.7%). Of the respondents, 89.0% reported factors that hindered counselling, of which customers’ characteristics (45.8%), the unavailability of medicines and other availability issues (32.5%), the features of interchangeable medicines (22.6%) and time pressure in the pharmacy (22.1%) were the most commonly reported. The benefits of the systems focused on cost savings for customers and society (74.4%). The most commonly reported problems concerned medicine availability (31.9%), changes in medicine prices and in reference price band (28.9%), as well as how GS is time-consuming and increases workload (24.2%).

**Conclusions:**

Finnish dispensers reported more hindering than facilitating factors in GS and RPS counselling. Customers’ characteristics were the most often mentioned in both cases. Customers’ knowledge could be increased by providing information and education. However, developing simpler regulations for GS and RPS, intelligent assisting software, and solutions for secured medicine availability would facilitate implementation of GS. Simplified price counselling would also guarantee the time needed and focus on instructions on the correct and safe use of medicines.

**Supplementary Information:**

The online version contains supplementary material available at 10.1186/s12913-022-08477-2.

## Background

Generic substitution (GS) and reference price system (RPS) are important ways to curb increasing pharmaceutical expenditures [1]. They are in use in most European countries. Although their implementation varies between countries, in most cases community pharmacies play the key role in implementing GS by recommending patients to substitute their medicine with a cheaper interchangeable medicine. The substitution is also often financially incentivized with a co-payment which is required if the patient declines to substitute the medicine. The final decision to substitute medicines, however, is made by the patient.

According to several studies, patients who have received information about GS or generics are more positive about them and more prepared to substitute their medicines [2–8]. Conversely, patients who have refused GS have deemed the information about GS they received insufficient [5]. Patients have also stated that lack of information is one of the reasons for not using generics [7, 9]. A lack of information about GS has also been reported to lead to confusion and suspicion, and even been associated with poorer medicine adherence [10, 11].

Pharmacists and physicians are common sources of information for patients about GS and generics [2, 4, 7, 11–13]. Patients value their opinions, and their recommendations have been one of the reasons for accepting GS [4, 7, 9, 14–16]. In several studies however, patients have more often received information about GS and generics from their pharmacists than their physicians [2, 4, 5, 11–13]. This highlights the importance of GS counselling at the pharmacy.

Although several studies have investigated counselling about medicines in pharmacies [17–22], only a few have focused on counselling about GS and interchangeable medicines [23–26], while other studies have presented some rather individual observations about counselling [11, 27, 28]. Based on these studies, it seems that the presence of GS increases the counselling time spent on non-medical issues, but not the total time of the encounter [24]. Most of the discussions about GS and interchangeable medicines include the possibility of substitution, medicine prices and price differences [23, 25, 26]. However, the most common questions customers ask about GS in the pharmacy relate to the similarity and equivalence of interchangeable medicines [25]. The information customers received about GS in pharmacies is usually considered sufficient [26]. Nevertheless, there is evidence that some customer characteristics like current use of medicines, age and education may influence the content of counselling.

Given the importance of the counselling, there is still a gap in research. To our knowledge, no research has been conducted into the factors affecting GS counselling and communication from the pharmacists’ point of view. In addition, the inclusion of a RPS in the counselling has rarely been taken into account in previous studies even though there are situations in counselling where GS and RPS are both included. Hence, the aim of this study is to explore dispensers’ perceptions of factors facilitating or hindering counselling about GS and RPS in community pharmacies. The aim is also to explore dispensers’ opinions about the benefits and problems of GS and RPS and to discuss them from the counselling point of view.

### Study context

In Finland, community pharmacies have been obliged to substitute a prescription medicine with the cheapest or close-to-cheapest interchangeable medicine since 2003 [29, 30]. In 2009 a RPS was introduced that includes medicines on the Finnish Medicines Agency’s list of interchangeable medicines and the national medicine reimbursement scheme [30–32]. In the RPS interchangeable medicines form a reference price group, and for each group a reference price is defined by adding €0.50 to the retail price of the least expensive interchangeable product of that group [32]. This €0.50 price difference is also known as the reference price band, and medicines within the price band are reimbursed based on their retail price. If the medicine is higher in price than the reference price and a customer declines to substitute the medicine, he or she must pay the excess in addition to the co-payment. Reference prices are valid for 3 months at a time. However, pharmaceutical companies can change the prices of their medicines every 2 weeks [33].

In community pharmacies, only pharmacists (M.Sc. in Pharmacy) and dispensers (B.Sc. in Pharmacy) are allowed to provide counselling on medicines [30]. They must ensure the customer knows how to use the medicine correctly and safely. Customers must also be informed about medicine prices, especially about the cheapest interchangeable product at the point of dispensing, as well as other factors affecting their choice of product. In addition, customers must be made aware that the interchangeable medicine being dispensed replaces the previously used product [34]. Dispensers (B.Sc. in Pharmacy) are the largest occupational group in pharmacies dispensing prescriptions and counselling customers about medicines, whereas pharmacists (M.Sc. in Pharmacy) usually work as supervisors alongside the pharmacy owner (M.Sc. in Pharmacy) [35].

## Methods

A postal questionnaire survey was carried out in February and March 2018 among dispensers (B.Sc in Pharmacy) working in Finnish community pharmacies. The random sample was formed from the membership register of the Finnish Pharmacists’ Association by choosing one-third of dispensers in the register (*n* = 1054). The decision to sample one-third of the dispensers was based on the previous survey study about Finnish dispensers [36]. The dispensers chosen received a questionnaire together with a cover letter and a prepaid return envelope to their home address. Two reminders were sent to each recipient. A response period of approximately 2 weeks was given in all mailing rounds.

The four-page questionnaire contained 18 questions (Additional file 1). The questionnaire was designed based on the legislative requirements set for the content of pharmacy customer counselling about GS [30, 34] and on some previous studies [37–41]. The questionnaire was tested for face validity by five academic colleagues who had experience in questionnaire surveys and were familiar with the study concept. The questionnaire was then tested by dispensers from two local pharmacies, with minor modifications made as a result.

This study reports the results of four open-ended questions regarding factors facilitating or hindering GS and RPS counselling and the possible benefits and problems of these systems. First respondents were asked what things make it easier to give advice to customers about GS and RPS, followed by a separate question asking what things make it more difficult. Respondents were also asked separately what in their opinion are the main benefits and the main problems of these systems. An open-ended question was also used to obtain background information on the respondents’ age, whereas gender, the location of the pharmacy and the number of prescriptions purchased per year in the pharmacy were the subject of structured questions. The first question of the questionnaire asked the respondents to state their current job at the pharmacy. Those respondents who were not currently working in a pharmacy were instructed to return the questionnaire blank.

According to the national ethical instructions for human sciences research, this study required no ethical review and the research process was in compliance with the guidelines [42]. Participation in the study was voluntary. Filling in the questionnaire and returning it to the researchers was considered as informed consent to participate.

### Data analysis

The questions were analyzed both qualitatively and quantitatively. In the qualitative analysis, responses to open-ended questions were analyzed using inductive content analysis. First, all responses to the question were entered in an Excel table (Version 2108, Microsoft corp). Responses were then broken down into analysis units that dealt with separate concepts. A unit could consist of a single word, phrase, or a group of sentences. If several different concepts were observed in the response, they were segregated into their own units. Analysis units were then simplified and sorted into subgroups. Similar subgroups were further merged into main groups, which were named to describe their content. This created an analytical framework for categories. The framework was drafted inductively from the responses until the saturation point was achieved. The point after which little or no new information emerged in the responses was considered as the saturation point for the question. The saturation points for factors facilitating counselling, factors hindering counselling, benefits, and problems of the systems were questionnaires 92, 159, 91, and 127, respectively. After the saturation point, the analytical framework was supplemented if new perspectives on categorization emerged in the rest of the responses. Each form was then examined, and responses were categorized according to the framework into the sub and main groups.

In all questions, the inductive content analysis was done by two researchers independently. The questions about the factors facilitating and hindering GS and RPS counselling were analyzed by a research colleague (PK) and the author RR, whereas the questions about the benefits and problems of GS and RPS were analyzed by authors EL and RR. The results were then compared with each other and contradictory categorizations were discussed until a consensus was made.

The categorization of responses was recorded in SPSS Statistics for Windows (Version 27; IBM Corp) for quantitative analysis. The data were further analyzed using frequencies and percentages. The respondents’ representativeness of the target population was analyzed using the Chi-square or Fisher’s exact test in terms of age and gender. Statistical significance was determined as *p*-values < 0.05.

## Results

Altogether 572 questionnaires were returned to the researchers. However, eight questionnaires were returned blank, and in 66 questionnaires dispensers reported they did not currently work in a community pharmacy. These questionnaires (*n* = 74) were therefore excluded, resulting in a final study sample of 980 dispensers and a response rate of 50.8% (*n* = 498). Dispensers aged ≤29 were overrepresented in the study compared to the target population (15.5% versus 11.4%, *p*-value = 0.009) (Table 1).

**Table 1:** Characteristics of the respondents and the target population

	Responding dispensers n (%)	Target dispensers n (%)^b^
*Gender*^*a*^	*n* = 493^c^	*n* = 3253
Female	466 (94.5)	3095 (95.1)
Male	27 (5.5)	158 (4.9)
*Age, years*^*a*^	*n* = 496^c^	n = 3253
≤ 29	77 (15.5)*	372 (11.4)*
30–39	133 (26.8)	889 (27.3)
40–49	133 (26.8)	994 (30.6)
50–59	121 (24.4)	841 (25.9)
≥ 60	32 (6.5)	157 (4.8)
*Number of prescriptions per year at the pharmacy*	n = 493^c^	
≤ 30,000	38 (7.7)	
30,001–60,000	76 (15.4)	
60,001–100,000	154 (31.2)	
≥ 100,001	225 (45.6)	
*Location of the pharmacy*	*n* = 492^c^	
Southern Finland	148 (30.1)	
Southwest Finland	58 (11.8)	
Western and Inland Finland	130 (26.4)	
Eastern Finland	88 (17.9)	
Northern Finland	48 (9.8)	
Lapland	20 (4.1)	

^a^Representativeness was analyzed in terms of the respondent’s age and gender

^b^Information based on the register of the Finnish Pharmacists’ Association in January 2018

^c^Some of the respondents did not report their gender, age, number of prescriptions per year at the pharmacy or the location of the pharmacy

* *p*-value = 0.009, Chi-square test

### Factors that facilitate GS and RPS counselling

Of the respondents, 75.9% (378/498) reported at least one factor that facilitates GS and RPS counselling in pharmacies. The most frequently mentioned factors concerned customers’ characteristics (36.5%), the information systems used in the pharmacy and their features (28.3%), and matters related to the features of interchangeable medicines (21.7%) (Table 2).

**Table 2:** Factors facilitating counselling about generic substitution (GS) and the reference price system (RPS)

**Main category**	**Respondents**^**a**^ **(*n*** **= 378)**
**Subcategory**	**n (%)**^**b**^
**Customers’ characteristics**	**138 (36.5)**
Awareness of GS and RPS	64 (46.4)
Interested in the subject, responsive to information, or a positive attitude towards GS and RPS	55 (39.9)
Ability to understand the GS and RPS counselling	25 (18.1)
Other (e.g. customer’s financial situation, age)	14 (10.1)
**Information systems used in the pharmacy and their features**	**107 (28.3)**
The information systems provide (clearly and easily) information on interchangeable medicines	71 (66.4)
Systems are easy to use, simple and function well	25 (23.4)
Other (e.g. computer screen helps to illustrate the counselling)	14 (13.1)
**Matters related to the features of interchangeable medicines**	**82 (21.7)**
Price (e.g. large price differences between preparations, stability of price changes)	59 (72.0)
Similar names of interchangeable medicines	8 (9.8)
Familiar or reliable manufacturer	6 (7.3)
Interchangeable alternatives for the medicine available within the reference price band	5 (6.1)
Other (e.g. the medicine is new to the customer, a local medicine brand)	13 (15.9)
**The medicine is available**	**61 (16.1)**
The medicine is in stock at the pharmacy	42 (68.9)
The medicine is available from a wholesaler	17 (27.9)
The medicine is available	13 (21.3)
**There is enough time for counselling and there is no hurry**	**61 (16.1)**
**The dispenser is professionally skilled**	**30 (7.9)**
Dispenser’s competence in GS and RPS (e.g. knowledge of GS and the RPS)	18 (60.0)
Dispenser can provide clear counselling	8 (26.7)
Dispenser recognizes the counselling as a legal obligation	7 (23.3)
Other (e.g. dispenser’s own activeness)	2 (6.7)
**Other**	**61 (16.1)**
Clear instructions for GS	8 (13.1)
Prescription-related subjects	8 (13.1)
Information from wholesalers	3 (4.9)
Other	42 (68.9)

^a^Respondents could list several factors; ^b^ Percentages of respondents in the main categories are calculated from the respondents to the question (*n* = 378), percentages of respondents in subcategories are calculated within its main category

Customers’ awareness, i.e. familiarity with GS and/or RPS as well as knowledge and experience of them were reported to facilitate counselling (Table 2). More detailed responses reported that the information received from the physician or featured in the media promoted awareness and counselling. Counselling was also reported to be facilitated by the customer’s interest in the subject, positive attitude, and receptiveness to counselling. The customer’s ability to understand was generally described as the ability to understand the system.

The information systems and their features as factors facilitating counselling were reported with particular emphasis on the pharmacy information system and its prescription processing features (Table 2). Respondents most commonly highlighted the importance of providing clear and useful information on interchangeable medicines supporting the counselling, such as prices and their differences, the reference price band, the range of interchangeable preparations and other properties of preparations such as whether or not the tablet could be split. Medicine prices and price differences were mentioned most often. Ease of use, and simplicity and good functionality were also mentioned.

Price, or more precisely large or clear price differences between interchangeable products, was most often reported to be a feature of an interchangeable medicine that facilitated the dispenser’s counselling (Table 2). Situations where prices remain stable and do not change much or often, and possible cost-savings for the customer were also mentioned as facilitating factors.

### Factors that hinder GS and RPS counselling

The majority of respondents (89.0%, 443/498) reported at least one factor that hinders GS and RPS counselling in pharmacies. The most frequently reported hindering factors dealt with customers’ characteristics (45.8%) and the unavailability of medicines and other availability issues (32.5%) (Table 3). Other frequently reported factors concerned matters related to the features of interchangeable medicines (22.6%) and time pressure in the pharmacy (22.1%).

**Table 3:** Factors hindering counselling about generic substitution (GS) and the reference price system (RPS)

**Main category**	**Respondents**^**a**^ **(*n*** **= 443)**
**Subcategory**	**n (%)**^**b**^
**Customers’ characteristics**	**203 (45.8)**
Difficulties in understanding GS and/or RPS	93(45.8)
Prejudice, lack of interest or negative attitude	57 (28.1)
Difficulties in communication	41 (20.2)
Prefer physician’s choice of the product	23 (11.3)
Unaware of GS or RPS	18 (8.9)
Not the user of the medicine	11 (5.4)
Other (e.g. age, illnesses, lots of medicines)	34 (16.7)
**Unavailability of medicines and other availability issues**	**144 (32.5)**
The medicine is not available in the pharmacy or from a wholesaler	122 (84.7)
Need to check the availability of the medicine from a wholesaler	18 (12.5)
Problems in availability after the change of reference price	13 (9.0)
**Matters related to the features of interchangeable medicines**	**100 (22.6)**
Small price differences between interchangeable medicines and informing customers about them	43 (43.0)
Different and difficult names of the interchangeable medicines	23 (23.0)
Large selection of interchangeable medicine options	23 (23.0)
Other (e.g. only one or small number of interchangeable medicines in the reference price band)	26 (26.0)
**Time pressure in the pharmacy**	**98 (22.1)**
**Changes in prices of interchangeable medicines, in the reference price band and its preparations**	**71 (16.0)**
Constantly/frequently changing prices	34 (47.9)
Changes in the reference price band and update of the new reference price	29 (40.8)
Variable position of interchangeable medicines in the reference price band	9 (12.7)
**Complexity of GS and RPS**	**57 (12.9)**
System is complex and difficult	36 (63.2)
System is difficult to explain clearly to the customer	13 (22.8)
Instructions and rules for GS and RPS are complex	12 (21.1)
**Shortcomings in the information systems used in the pharmacy**	**34 (7.7)**
The necessary information is not easily or sufficiently visible (e.g. price differences between medicines), or not found at all	29(85.3)
Other (e.g. the pharmacy system is complex)	6 (17.6)
**Other**	**52 (11.7)**
Matters related to dispensers (e.g. negative attitude, insufficient competence)	6 (11.5)
There are lots of things to tell the customer	4 (7.7)
Other	42 (80.8)

^a^Respondents could list several factors; ^b^ Percentages of respondents in the main categories are calculated from the respondents to the question (*n* = 443), percentages of respondents in subcategories are calculated within its main category

Customers’ characteristics that were reported to hinder counselling mainly concerned the customer’s difficulties in understanding, i.e. either they do not understand, or have trouble understanding, the system (Table 3). Detailed descriptions included examples such as a customer does not understand the reference price system or that the active substance is the same in interchangeable medicines. It was also often pointed out that older people have trouble understanding. Customers’ prejudice towards GS and interchangeable medicines, a lack of interest or a negative attitude were reported to hinder counselling. One in five respondents in the main category also reported customers’ difficulties in communication such as the lack of a common language or the customer’s poor hearing, as hindering factors.

Hindering factors related to the unavailability of medicines and other availability issues mainly dealt with situations when medicine was not available in a pharmacy or from a wholesaler (Table 3). The unavailability of medicines was reported almost as often from the perspective of both pharmacies and wholesalers. In addition, the need to check the availability of the medicine from a wholesaler, its difficulty, and how it takes time were reported to hinder counselling. There were also reports that a change in reference price was considered to hinder counselling, as cheaper interchangeable preparations belonging to the reference price band may be out of stock from the pharmacy or wholesalers.

Small price differences between interchangeable medicines and informing customers about these differences were the most frequently reported features of interchangeable medicines that hinder counselling (Table 3). Some respondents mentioned that informing customers about very small price differences takes time, feels foolish or may frustrate the customer. Different and difficult names of interchangeable medicines were mentioned as hindering factors and further reported as possibly confusing for some customers. A large number of interchangeable medicine options was also reported by some respondents as making it difficult for dispensers to introduce available options and for customers to decide what to choose.

### Benefits and problems of GS and RPS

The vast majority of respondents reported at least one benefit and one problem regarding GS and RPS (95.6% (476/498) and 93.8% (467/498), respectively). The most frequently reported benefit was cost savings for customers and society (74.4%) (Table 4). Other commonly reported benefits were lower medicine prices and price competition (22.3%) and the better security of medicine supply in the event of availability problems (19.3%). Correspondingly, when reporting problems with GS and the RPS one in three respondents reported medicine availability problems (31.9%) and changes in medicine prices and reference price band (28.9%). One in five respondents reported problems with how GS takes time and increases workload (24.2%).

**Table 4:** Benefits and problems of GS and RPS as reported by the dispensers

**Benefits**	**Respondents** **(*n*** **= 476)**^a^ **n (%)**
Cost savings for customers and society	354 (74.4)
Lower medicine prices and price competition	106 (22.3)
Improved availability in medicine shortages	92 (19.3)
Customer has the right to decide on GS and the freedom to choose the product	65 (13.7)
Improved medication adherence by choosing the most suitable preparation for the customer^b^ and due to a lower medicine price	33 (6.9)
No need to contact the physician about GS	25 (5.3)
Several optional interchangeable medicines	13 (2.7)
Other^c^	36 (7.6)
**Problems**	**Respondents** **(*n*** **= 467)**^a^ **n(%)**
Medicine availability problems	149 (31.9)
Changes in medicine prices and reference price band	135 (28.9)
GS takes time and increases the workload (e.g. price counselling, explaining the system to customers, checking the availability of interchangeable products)	113 (24.2)
Inventory control is challenging (e.g. not everything or even the cheapest can be kept in stock, difficulty in storage due to the unpredictability of preparations within reference price bands)	101 (21.6)
Change of the preparation due to GS may pose a risk to medication safety (e.g. changes in name, packaging or appearance confuse customers, double medication, complications in keeping up to date with medicines in use)	100 (21.4)
The system is complex for the customer to understand	72 (15.4)
Actions by pharmaceutical companies (e.g. setting really low prices for a small batch of medicines)	54 (11.6)
Customers’ suspicious or negative attitude towards GS	42 (9.0)
Obligation to inform the customer about the cheapest product is frustrating (e.g. because of small price differences between products)	33 (7.1)
Differences between interchangeable preparations (e.g. experienced efficacy, side effects, whether or not tablets can be split)	30 (6.4)
Information systems problems (e.g. do not provide enough information about availability)	19 (4.1)
Too many interchangeable medicines options	18 (3.9)
Other^d^	68 (14.6)

^a^Respondents could list several factors; ^b^e.g. in terms of excipients, whether or not tablets can be split, packaging; ^c^e.g. no need to have every interchangeable preparation in stock in the pharmacy, customer satisfaction; ^d^Lower profitability and margins for pharmacies, contraceptive pills are not interchangeable. There may be a large cost to the customer if substitution is declined

When reporting cost savings as a benefit, respondents more frequently considered it from the customer’s perspective rather than society’s (Table 4). Lower medicine prices and price competition were considered to be favorable results of GS and RPS. Respondents reported that medicine prices are now lower and reasonable, often mentioning price competition as a cause. Prices were also reported to have remained reasonable.

GS and the RPS were considered to improve customers’ access to medicines as they could be provided with another interchangeable medicine if the pharmacy or wholesaler was out of stock of the prescribed product (Table 4). Some respondents also stated that because of GS the security of medicine supply is now better.

When asked about problems connected with GS and the RPS, medicine availability problems were said to be problematic in general, in terms of the cheapest products, and after a change in reference price (Table 4). The cheapest products were often reported to be out of stock by wholesalers. In addition, medicine shortages were reported to be likely soon after a change in reference price due to the small number of products within the reference price band, as they would quickly go out of stock by wholesalers.

Respondents considered that changes in medicine prices and in reference price bands are problematic and that they change too often (Table 4). A change in the reference price band was often reported to result in a small number of interchangeable products within the band for the following 2 weeks. The narrow reference price band of €0.50 was also generally considered problematic.

Respondents reported that GS could be time-consuming and increase the workload in the pharmacy (Table 4). Respondents most frequently reported that price counselling takes too much time. Explaining matters related to GS or the RPS to the customer and checking the availability of the medicine were also considered time-consuming. Many respondents highlighted the fact that the time spent would reduce the time available for medicine counselling itself. GS was reported to increase the workload, e.g. in counselling and inventory maintenance after major changes in the reference price band.

## Discussion

In this study, dispensers reported factors that hindered GS and RPS counselling more often than those that facilitated it. Customers’ characteristics were most commonly reported as both hindering and facilitating factors. Counselling was also often considered to be hindered by the unavailability of medicines and other availability issues. Information systems were often reported as a facilitating factor by providing counselling with supportive information about interchangeable medicines. More topics were reported on the problems of GS and the RPS than benefits. The benefits of these systems mainly focused on the cost savings for customers and society, whereas problems were fairly evenly distributed among several topics, of which the most often reported dealt with medicine availability problems, changes in medicine prices and reference price band, along with how GS takes time and increases workload.

According to this study, it appears that customers are one of the key factors affecting the fluency of GS and RPS counselling. In particular, customers’ knowledge of GS and the RPS and their understanding of these systems seems to matter. If the customers are unfamiliar with the systems, it may be difficult for them to understand the counselling and what is happening during GS. The counselling may then focus more on the customer’s potential questions about the systems, and thus the whole counselling process might put the emphasis on explaining the systems. The other results of this study indicate that this might happen at the expense of medicine counselling. In this study, dispensers reported that explaining the systems to customers takes time and is one of the reasons for the time consumption and increased workload of GS and RPS counselling. Less time was also reported to be available for medicine counselling itself.

Results supporting this theory have been found in other studies [24, 43, 44]. A Swedish study found that the presence of GS increased the time spent on non-medical counselling, without prolonging the counselling situation [24]. In another study, pharmacists reported that since the introduction of GS patient-pharmacist communication had started to be dominated by topics such as money, equivalence, packages and the color of medicines instead of counselling about treatment or medications, for example [43]. In addition, the lack of time to provide patients with the relevant information has been perceived by pharmacists as a challenge in implementing generic medication use [44].

Thus, it is important to inform customers about GS and the RPS in order to provide smooth counselling and to be able to spend more time on medical counselling. However, occasional information campaigns are not enough; information should be continuous, as there will constantly be new medicine users to be introduced to the systems. Information should also focus on issues that are unclear to customers, for example the equivalence of interchangeable medicines, which in a previous study was one of the most common questions presented to dispensers by customers [25]. The people in need of information should be identified. According to research, customers who have no prior experience of GS or who have refused GS, have considered the information received insufficient [5, 25]. In some studies, pharmacists have also raised concerns that older people find it difficult to understand the concept of substitution [10, 43].

Customers’ difficulties in understanding GS have caused pharmacists to be concerned that the substitution could confuse the customer, and even lead to medication errors and double medication [43]. Factors that confuse customers have included differences in the names, appearance, and packaging of medicines [4, 6, 45–47]. Similar results were also found in this study. The different names of interchangeable medicines were reported to complicate counselling and possibly even to confuse customers, while similar names facilitated counselling. According to Finnish regulations, the dispenser must ensure that the customer is aware that the interchangeable medicine being dispensed replaces the previously used product [34]. Thus, it is logical that the similarity of names will facilitate counselling, as it might prevent confusion by providing clarity to the customer when the packaging or appearance of the medicine changes. From the point of view of counselling, attention should thus be paid to factors that prevent possible confusion of the customer. In addition, the effects of GS and related counselling on medication safety should be studied in the future.

The system should be simplified to make it clearer to the customer and easier for pharmacists to execute. In a Portuguese study, for example, pharmacy professionals considered it difficult to explain the frequent change in prices to customers [48]. Similar findings were also made in this study, as continued price fluctuations and changes in the reference price band were reported to complicate counselling. They were also identified as one of the most common problems with the systems.

In this study, counselling was reported to be facilitated by pharmacy information systems as they provide information about interchangeable preparations, which supports counselling. This was especially the case when talking about prices and price differences. Price counselling is an essential part of GS and the RPS, since the desire to save on costs is one of the reasons why customers substitute their medicine [7, 9, 36, 37, 49, 50]. Thus, the ease and fluency of price counselling can be considered relevant for the cost saving potential of GS and RPS. In terms of medicine prices, large price differences in preparations and price stability were reported to facilitate counselling. On the other hand, price counselling was reported to be time consuming, leaving less time for medicine counselling. It would therefore be wise to seek ways to facilitate price counselling. One option could be to prolong the validity of medicine prices, with prices fluctuating less frequently.

The potential of pharmacy information systems to respond to factors that complicate the counselling should also be examined. Based on this study, good accessibility of medicine availability information could improve the counselling workflow. Above all, digital technologies should support pharmacists in counselling, as pharmacists are an important source of information for customers and support their decision-making [2, 4, 5, 9, 11–16].

The availability of medicines is also an important factor influencing counselling, as the unavailability of medicines from pharmacies or wholesalers was the single largest subcategory of factors complicating counselling. When describing problems with the systems, dispensers raised concerns about medicine availability, particularly that of cheaper alternatives. Availability problems were also often reported to occur after bigger changes in reference prices. On the other hand, counselling was clearly facilitated when substitutable medicines were in stock in the pharmacy. This is natural, as it is easier to suggest a preparation that is in stock, which means the customer can obtain it right away. The storage situation has in fact been the most common criterion by which dispensers choose an interchangeable preparation to offer to the customer [51]. Solving the availability problems of cheaper medicines, especially after a bigger change in reference price, could thus facilitate counselling. This could be done, for example, by obliging pharmaceutical companies to provide a sufficiently large medicine batch as a condition for their product to be included in the reference price band.

### Strengths and limitations

This study provides new information about GS and RPS counselling and the factors that facilitate and hinder it. It also gives an insight into the benefits and problems of these systems from the counselling perspective. In Finland, GS and the RPS have been in use since 2003 and 2009, respectively. Thus, dispensers are well experienced with them and are the right target group to answer practical questions about counselling of these systems and about their benefits and problems.

The respondents in this study were obtained from the register of members of the Finnish Pharmacists’ Association, which comprises the vast majority of dispensers working in community pharmacies in Finland. The respondents well represented the target population except for respondents/dispensers aged ≤29 years, who were slightly overrepresented in the study.

The sample size of the study was large (*n* = 498) and the response rate of 50.8% is similar to, or higher than, that in other GS postal surveys for pharmacists, where response rates have ranged from 15 to 58% [39, 52]. In addition, the adequacy of the data for quantitative analyses was assessed afterward by sample size calculation. The calculation resulted in a sample size of 344 dispensers (95% Confidence level, 5% margin of error, *N* = 3253). Thus, the amount of data can be considered adequate for quantitative analysis. The response rates for the questions reported in this study were high (75.9–95.6%), which suggests that the questions were probably understandable.

The use of open-ended questions in this study made it possible to gain a broad insight into the research topics. However, responses to open-ended questions can sometimes be open to interpretation and, unlike in interview studies, surveys cannot request clarifications to responses. However, the qualitative analysis of the questions in this study was done by two researchers independently, which makes the results more reliable. In addition, the material in our study was quite large, which permitted a good and fairly reliable evaluation of the factors affecting counselling.

It must be noted that the data of the study was collected in spring 2018. Thus, it is already 4 years old. However, no changes have been made to the legislation on GS or RPS in Finland after 2018. The implementation of GS in pharmacies and the principles of its counselling have remained substantially similar, and therefore the results of this study can still be considered relevant.

## Conclusion

Finnish dispensers reported more hindering than facilitating factors in GS and RPS counselling. Customers’ characteristics were the most often mentioned in both cases. In particular, customers’ knowledge of GS and the RPS, and their ability to understand counselling seemed to play a role in the fluency of counselling. It is therefore important to improve customers’ knowledge through information and education. Information systems used in pharmacies were considered to facilitate counselling by providing information on interchangeable medicines. On the other hand, medicine availability problems were reported to hinder counselling and were seen as the most common problem of GS and the RPS. GS was reported to take time and to increase workload, for example in cases of price counselling and explaining the system to the customer, leaving less time for medicine counselling. Thus, developing simpler regulations for GS and the RPS, intelligent assisting software and solutions for secured medicine availability would facilitate implementation of GS. Simplified price counselling would also guarantee the time needed and focus on instructions on correct and the safe use of medicines.

## Supplementary Information

**Additional file 1.**

## Data Availability

The datasets generated and analyzed during the current study are not publicly available because permission has not been sought from respondents to share material. The data that support the findings of this study may be available from the corresponding author on reasonable request.

## Abbreviations

GS: Generic substitution
RPS: Reference price system
